# Supplementary material for: Are carbon emissions trading and green financial instruments synergistic? -Comprehensive quantitative research based on content analysis
Source: PLoS One. 2024 Mar 7;19(3):e0298601. doi: 10.1371/journal.pone.0298601 (PMC10919601; doi:10.1371/journal.pone.0298601)
Supplement: S1 Data — (ZIP) [file pone.0298601.s001.zip › Data/地级市Z_F工作报告.zip!/╕ú╜¿/╞╬╠∩╩╨/└┤╫╘╣1⁄2╓┌║┼í╛┬φ┐╦╩2╛▌═°í┐_2017.docx]

各位代表：

　　现在，我代表莆田市人民政府，向大会报告政府工作，请予审议，并请市政协委员提出意见。

　　一、2016年和本届政府工作回顾

　　过去一年，在省委、省政府和市委的坚强领导下，市政府团结依靠全市人民，认真贯彻党的十八大和十八届三中、四中、五中、六中全会精神，深入贯彻习近平总书记系列重要讲话精神和对福建工作的重要指示，按照建设“机制活、产业优、百姓富、生态美”的新福建要求，围绕“坚持绿色发展，打造宜居港城、建设美丽莆田，创建美丽中国的示范区”奋斗目标，扎实做好各项工作，基本完成市六届人大六次会议确定的各项目标任务。

　　初步预计，地区生产总值1811亿元、增长8.9%；一般公共预算总收入180.1亿元、下降2.8%，地方一般公共预算收入115.7亿元、增长0.1%；全社会固定资产投资2000亿元、增长13%；外贸出口总额197亿元、增长0.5%，实际利用外资4亿美元、增长6%；社会消费品零售总额626亿元、增长12%；居民消费价格总水平上涨1.5%；居民人均可支配收入2.3万元、增长8%；城镇登记失业率2.19%；年度节能减排任务全面完成。

　　一年来的主要工作和成效是：

　　（一）积极应对下行压力，经济平稳健康发展。坚持稳增长调结构促转型，落实国务院和省政府降低实体经济企业成本等系列政策，减轻企业负担20多亿元，筹集各类扶持资金7.8亿元，实施技改项目167项，帮助企业转贷115.6亿元，处置不良贷款36亿元，稳住工业大盘，实现规模以上工业增加值845亿元、增长8.4%。积极发展现代服务业，加快房地产去库存，促进第三产业多作贡献，实现增加值666亿元、增长12.2%。加快发展现代农业，实现农业增加值125亿元、增长1.2%。持续扩大有效投资，推进“五个一批”项目，468个重点项目实现投资946亿元，开工68个、竣工62个。

　　（二）统筹城乡协调发展，基础设施日臻完善。城建投资845亿元、增长9%，新建改建城市道路98条、绿道63公里、燃气管道20公里，新增一批停车场、公交线路和公园。新建及扩容污水处理厂3座、污水管网216公里，生活和医疗垃圾日处理能力达1800吨。水利设施投资85亿元、增长38.1%，续建新建47个重大水利项目，交通设施投资250亿元，改进和创新出租车运营管理，完成城乡客运公交化改造和部分陆岛交通码头扩建，自然村道路硬化率达92%，涧口、后黄等新型农村社区建设有效推进。

　　（三）纵深推进改革开放，市场活力不断激发。改革红利持续释放，取消审批事项24项，下放14项，新增市场主体4.6万户，增长18.8%，食品药品“九证合一”改革成为可复制推广样板。实行国企分类管理，全面落实“营改增”工作，三棵树成功上市，2家企业在新三板挂牌，完成机关事业单位养老保险改革，农村产权制度和不动产登记制度改革有序推进。成功举办首届世界妈祖文化论坛，发出《湄洲倡议》。

　　（四）有效增进民生福祉，社会事业稳步发展。民生投入162亿元、增长13%。18项为民办实事项目基本完成。城镇新增就业2万人，转移农村劳动力3万人，造福搬迁1万人，脱贫1.6万人。城乡居民基础养老金和基本医疗保险人均财政投入分别达1100元和420元。新建幼儿园14所、增加学位3500个，新建中小学校舍37幢、增加学位8000个，千人均病床位达4.8张、卫技人员达4.79人，二孩政策全面实施。列入“海丝”申报世界文化遗产联盟城市，举办第25届世界技巧锦标赛等赛事，广泛开展群众性文体活动。安全生产、社会综合治理平稳向好，成功防御“尼伯特”“莫兰蒂”“鲇鱼”等台风。

　　（五）切实转变工作作风，提升政府服务水平。扎实开展“两学一做”学习教育，推行“马上就办”，办理审批、服务事项150多万件，做好项目用地、用海、用林保障。坚持依法行政，推进政务公开、政府信息公开，主动接受人大、政协和社会监督，加强与法院、检察院联系互动，办理市人大建议159件、市政协提案247件，办结率均为100%。基本完成公务用车改革，党风廉政建设和反腐败工作扎实推进。

　　各位代表，市六届人大一次会议以来，我们走过极不平凡的五年。这五年，综合实力显著壮大，城乡面貌显著变化，人民生活显著改善，改革活力显著增强，作风建设显著加强，为继续前进打下坚实基础。

　　五年来，我们围绕培育大产业，以新理念引领新常态，推进供给侧结构性改革，加快转变发展方式，综合实力显著壮大。预计地区生产总值是五年前的1.7倍，年均增长11.2%。一般公共预算总收入年均增长11.8%、地方一般公共预算收入年均增长12.6%，总量均从全省第7位提升至第6位。全社会固定资产投资累计7339亿元，年均增长22.5%。第一产业增加值年均增长2.6%，产值超亿元农业龙头企业达40家，实施“336”工程，五年开工建设重大产业类项目156个，竣工投产114个，规模以上工业企业增加值年均增长12.8%，总量从全省第6位提升至第5位，新增产值超10亿元企业30家，第三产业增加值年均增长11.8%，总量从全省第7位提升至第6位，三次产业比例从9.4：58.4：32.2调整为6.9：56.3：36.8，节能减排降碳任务全面完成。莆田高新区升级为国家级高新区，建成鞋业研发设计中心、鞋类检测中心、浆纸产品质检中心等一批国家级平台和企业技术中心，研发经费投入占地区生产总值比重提高到1.3%，万人发明专利拥有量从五年前0.2件提高到1.85件，荣获全国科技进步城市“五连冠”。

　　五年来，我们围绕建设大城市，加快基础设施和生态文明建设，率先推进新型城镇化和城乡一体化试点建设，城乡面貌显著变化。认真实施《莆田市城乡一体化综合配套改革试验总体方案》，城市建成区面积87平方公里，比五年前增加34平方公里，城镇化率57.8%，从全省第6位提升至第4位。建成万吨级以上泊位7个，新增港口吞吐能力2554万吨，东吴港区列入全国重要港区名录，向莆铁路和湄洲湾港口铁路东吴支线建成通车，日发送旅客1.6万人次，铁路货运实现零的突破，高速公路形成“两纵两横”网络，通车里程是五年前的3.8倍，“三纵一横三联”国省干线全面建设，公路网密度居全省第一，完成城市道路“白改黑”166公里，宽带光纤基本实现全覆盖。建成仙游抽水蓄能电站、金钟水利枢纽引水配套工程、东圳大坝除险加固工程、木兰溪下游防洪工程，整治内河180公里，解决126万农村人口饮水安全问题，新建扩建污水处理厂6 座、城乡污水管网1700公里。农村就地城镇化和就近市民化步伐加快，特色小镇、新型农村社区、幸福家园、美丽乡村等试点走在全省前列。城乡生态更加清新，森林覆盖率达59.8%，绿地覆盖率达38.8%，人均公园绿地面积达12.7平方米，绿道总里程达220.5公里，空气质量连续四年居全省第一，荣获全国绿化模范城市、国家园林城市等荣誉称号。

　　五年来，我们围绕保障大民生，坚持共建共治共享，把发展成果更好体现在增进民生福祉上，不断加大民生投入，人民生活显著改善。70%以上的财政资金用于民生支出。实施78项为民办实事项目，新增城镇就业11.1万人，转移农村劳动力21.5万人，居民人均可支配收入是2011年的1.6倍，率先在全省实现城乡居民养老保险一体化、医疗保险一体化，社保五险覆盖率稳步提高，城乡低保应保尽保，农村低保标准五年提高5280元。精准扶贫持续推进，造福搬迁6.3万人，脱贫7.1万人，实施保障性安居工程，建成6万套保障房。所有县区通过国家义务教育发展基本均衡认定，教育信息化走在全省前列，莆田学院、湄洲湾职业技术学院分别通过教育部本科教学、高职高专人才培养工作水平评估。医药卫生体制改革稳步推进，组建市级医疗集团，实行药品耗材零差率和分级诊疗，率先在全国实行医师多点执业，坚持计划生育基本国策。城乡文体设施得到改善，新增110多个市级以上文物保护单位和非遗项目，木兰陂列入世界首批灌溉工程遗产名录，湄洲妈祖祖庙入选“海丝”申遗首批遗产点名单，成功举办第五届亚洲体操锦标赛等赛事，广泛开展全民健身活动。防汛救灾机制更加科学高效，平安莆田建设持续深化，安全生产主要指标连年下降，获得全国文明城市提名，荣膺全国双拥模范城“五连冠”。工会、共青团、妇联、档案、地方志、残疾人、红十字等工作取得新进步。

　　五年来，我们围绕激发大活力，直面制约发展的矛盾和问题，努力开拓创新、先行先试，重点领域和关键环节改革不断突破，对外开放水平不断提高。新型城镇化、电子商务、信息惠民、智慧城市、民营经济、新能源、知识产权、防震减灾等8个国家级改革试点有序开展。行政审批改革深入推进，商事制度改革红利效应显著，市场主体增加到23.8万户，是五年前的3.2倍，总量从全省第9位提升至第5位。市属国有企业改革重组，实现监管全覆盖和资产保值增值，总资产达604亿元，是五年前的4.3倍。加快财税体制改革，全面推开“营改增”，设立基础设施基金、产业基金、产业互助成长资金，争取专项债券基金30.8亿元、专项建设基金58亿元。对外开放交流持续深化，妈祖文化写进国家“十三五”规划纲要，上升为国家战略，莆港澳台侨密切交流，湄洲岛列为“中国华侨国际文化交流基地”，与马来西亚诗巫市、澳大利亚帕拉马塔市缔结友好城市。

　　五年来，我们围绕法治政府、创新政府、廉洁政府和服务型政府建设，加快职能转变，提升服务水平，努力建设为人民服务、让人民满意的政府。扎实开展党的群众路线教育实践活动、“三严三实”专题教育和“两学一做”学习教育，践行“马上就办、真抓实干”要求，严格落实党风廉政建设责任制，认真落实中央八项规定精神，驰而不息纠正“四风”。坚持依法行政，自觉接受监督，落实向人大报告和向政协通报工作的常态化机制，累计办理市人大代表建议918件、政协提案1244件，办结率均为100%。加快政府职能转变，精简机构9个，承接审批事项94项、下放243项、取消124项、调整33项，政府执行力、公信力全面提升。

　　各位代表，五年的成绩来之不易，这是省委、省政府正确领导和关心支持的结果，是市委坚强领导，全市上下团结拼搏，社会各界人士和海内外朋友鼎力支持的结果。在此，我代表市人民政府，向全市人民，向各民主党派、各人民团体和社会各界，向驻莆部队、武警官兵，向港澳台同胞、海外乡亲和国际友人，表示衷心的感谢！

　　在看到成绩的同时，我们也清醒地认识到存在的不足和问题，主要是：经济总量偏小，财政收入增长放缓，投资力度减弱，重大项目储备不足。产业转型升级的基础还不牢固，部分企业生产经营困难，金融风险防控化解任务繁重。城市防洪排涝、地下管网等设施建设存在短板。城乡发展存在差距，脱贫攻坚任务依然艰巨。优质教育、优质医疗和公用设施总量不足，城乡公共服务水平有待提升。一些干部创新意识、担当能力、服务理念、作风养成还不适应新要求，营商环境有待进一步改善。对此，我们务必高度重视，认真解决。

　　二、今后五年工作的总体要求和目标任务

　　今后五年工作的总体要求是：高举中国特色社会主义伟大旗帜，以马克思列宁主义、毛泽东思想、邓小平理论、“三个代表”重要思想、科学发展观为指导，深入贯彻习近平总书记系列重要讲话精神和对福建工作的重要指示，紧紧围绕“五位一体”总体布局和“四个全面”战略布局，牢固树立和落实五大发展理念，按照“再上新台阶、建设新福建”的要求，认真落实市第七次党代会部署，以保持经济稳定较快增长为目标，以转型升级为主线，以提高发展质量和效益为中心，把发展临港先进制造业作为支撑，实施“336”工程，坚持绿色发展，加快形成引领经济发展新常态的体制机制和发展方式，全面推进经济建设、政治建设、文化建设、社会建设、生态文明建设和党的建设，努力打造宜居港城、建设美丽莆田，创建美丽中国的示范区。

　　今后五年奋斗目标是：经济保持稳定较快发展。主要经济指标增幅高于全省平均水平1-2个百分点，到2021年，地区生产总值超过3000亿元，人均地区生产总值超过全省平均水平，一般公共预算总收入达300亿元以上。产业体系更加绿色低碳。以创新驱动为导向，供给侧结构性改革取得成效，产业布局和产业结构加速绿色低碳转型，经济发展的质量和效益明显提高，单位地区生产总值二氧化碳排放比2015年下降18%以上。城市发展更加系统协调。按照中心城区人口超百万的思路，加快建设“大城市”，城市空间布局进一步优化，城市发展的系统性、协调性、生长性，承载力和辐射力显著增强。人居环境更加宜居舒适。城乡基础设施更加完善，“一村一韵”“一村一景”独特风貌逐步彰显，生态环境质量持续保持全省前列，生态文明示范区加快建设，建成全国森林城市。人民生活更加幸福和谐。全市居民人均可支配收入比2010年翻一番。贫困人口全部实现脱贫，基本公共服务均等化程度居全省前列。文化教育水平、医疗健康水平、民生保障水平、市民素质和社会文明程度显著提高，社会治理体系更加完善，人民群众安居乐业，成为全国文明城市，全面建成小康社会。

　　各位代表，市第七次党代会绘就了“坚持绿色发展，打造宜居港城、建设美丽莆田，创建美丽中国的示范区”的宏伟蓝图，《建设美丽莆田行动纲要》目标振奋人心。我们坚信，在市委的坚强领导下，只要我们对标看齐，奋力赶超，全力实施“一区、一中心、五品牌、九行动”，就一定能够实现跨越发展；只要我们敢于担当，锲而不舍，一步一个脚印，就一定能够把美好蓝图变成现实。

　　今后五年，我们要高端定位，构建绿色低碳的现代产业体系。实施绿色产业专项行动，注重招商选资，推动产业向中高端迈进。加快发展现代农业，推进农业供给侧结构性改革，推动适度规模经营，创新农村金融，培育品牌农业、智慧农业、生态农业、现代渔业，促进一产“接二连三”融合发展，培育一批产值超10亿元农业产业化龙头企业，打造全国生态海洋牧场示范基地。全力打造十大优势产业，对标先进制造地区，完善产业培育机制，做到每一个产业，都有一个发展规划、一支产业基金、一套管用政策、一个咨询团队、一个创新平台、一个推进机制。支柱产业，重点扶持有市场有优势的鞋服、工艺美术、食品企业，通过兼并重组、联盟创新、联合创牌实现转型升级，积极引导莆商、莆资、莆智回归发展特色产业，全面培育全国一流的鞋艺、工艺、银饰、珠宝、油画、啤酒文化等特色小镇，打造工艺美术、绿色食品国际品牌；同时，重点发展合成纤维、粘胶纤维、纺织新材料等化工（纺织化纤）产业链项目，培育精细化工终端产业集群，打造东南沿海重要的纺织化纤新材料基地。新兴产业，重点引进一批带动性强的电子信息、高端装备和海洋新兴产业链项目，打造我国重要的新型显示和集成电路产业基地；抢占健康医疗产业制高点，全力打造面向全国的集高端专科医院集群、健康养生旅游中心、先进医药器械制造基地、民营医院总部、医疗人才培训基地、药械交易平台、医疗产业金融平台于一体的健康医疗小镇，打响医疗健康国际品牌。其它重点产业，积极推广绿色建筑，大力发展建筑产业总部经济，推动建筑产业做大做强；加快发展清洁能源，做大能源产业，打造国家级LNG大型战略储备基地、全国大型海上风电示范基地。全面构建产业生态圈，实施开放创新专项行动，承接新一轮国际高端服务业转移，加快发展现代物流、电子商务、金融服务、创意设计等，建设科技服务、跨境电商、物流信息等平台，建设创新基地和众创空间，引进一批创业投资机构，创新服务业态，形成一批省级以上服务业集聚区，建成全国知名的电商集聚区和跨境电商示范区，到2021年，规模以上工业企业增加值1500亿元，服务业增加值占地区生产总值比重达42%以上。

　　今后五年，我们要持续领先，打造生态清新的绿色家园。对标现代生态都市，实施生态文明专项行动，严守生态保护红线，落实最严格的环境保护制度，深化城乡污染防治，加强生态修复，留住青山绿水、碧海蓝天。建好城市绿心，加强规划和管控，按照生态绿心、文化绿心、和谐绿心的定位，全面推进城市绿心的整治和建设，打造莆田城市新名片。建设森林莆田，实施“四绿”工程，完善提升城市绿色屏障，建设景观丰富、层次分明、生态环境优美的森林通道，加快城市绿化美化，新建一批公园，建成莆田植物园，形成森林围城的空间格局。打造生态水系，加强东圳水库等重大水源地保护，加强木兰溪、萩芦溪流域、南北洋及城区河道治理，建成木兰溪全线防洪工程，统筹布局一批人工湖，建设集防洪、绿廊、景观、休闲于一体的南北洋生态水系。保护海洋资源，保护海洋生物物种多样性，合理开发利用滩涂、海岛以及岸线资源，建设滨海生态走廊。健全生态文明体制，完善流域上下游对等补偿机制，建立生态公益林补偿机制，推行碳排放权交易制度，加大对上游地区转移支付力度，2021年前，建成全国水生态文明试点城市、国家森林城市、国家生态城市，生态环境质量持续保持全省前列。

　　今后五年，我们要创新示范，推进城乡一体的新型城镇化。对标发达城市，用系统的思维，全力实施城乡建设专项行动，构建“一主两副”城市格局。做美做大中心城区，遵循自然山水格局，坚持留白、留绿、留脉，突出宜居、宜业、生态，精致打造环绿心大都市，建设魅力之城、生态之城、创业之城、文明之城，到2021年，城市建成区面积扩大到120平方公里以上，跨入超百万人口大城市行列。加快组团联动发展，城市东进南扩，东进建设大学城片区，面向兴化湾打造先进制造业基地。南扩发展木兰溪南岸新区、高铁站片区，联动发展滨海新城和仙游县城，建设木兰溪南岸高端现代服务业集聚区、水绿交融的土海片区、面向全国的健康医疗小镇、文化浓郁的湄洲岛和妈祖城。构建通达便利交通体系，建设福厦客专、福莆宁城际铁路,争取建设莆田机场和城市轨道交通，推进沈海高速扩容工程，构建绕城高速，建设连接城市组团的快速通道，完善城市主次干道网络，畅通市内支路微循环，实现城区15分钟上高速、镇镇20分钟上高速。推动城乡和谐交融，深化国家新型城镇化综合试点和全省城乡一体化综合配套改革试点，建设镇级小城市、中心镇街、新型社区和美丽乡村，传承乡愁记忆，留住田园风光，享受都市生活，激活农村活力。继续支持仙游山区、北高、埭头半岛等薄弱区域加快发展。到2021年，城镇化率达68%左右。

　　今后五年，我们要勇于超越，建设面向国际的亿吨大港。对标国际航运中心，创新港口发展模式，实施基础设施专项行动，打造深水良港国际品牌。建大港设施，修编实施湄洲湾港总体规划，续建新建一批深水泊位和港航设施，建成我国南方最大的散货码头和湄洲湾40万吨主航道，完善港口货运铁路系统，提升疏港公路运输能力。兴大港业态，规划建设港口航运总部经济区，建立矿石、煤炭、粮食、木材等大宗货物区域分拨中心，建立国家级港口航运物流发展平台，发展国际邮轮、客货滚装等业态，打造集货代船代、航运信息、航运交易、期货交割、金融保险等高端服务于一体的港区生态圈。创大港品牌，融入“一带一路”建设，发挥区位优势，推动莆台港口深度合作，建设“21世纪海上丝绸之路”的物流和贸易中心。组建面向长江中下游和粤西地区的港口战略联盟，依托向莆铁路对接“陆地港”和“飞地港”，打造内陆地区重要的出海口。到2021年，港口通过能力突破1亿吨。

　　今后五年，我们要传承文脉，提升莆仙文化的软实力。建成世界妈祖文化中心，把传承和弘扬妈祖文化融入“海丝”战略，持续办好世界妈祖文化论坛，提升论坛内涵，建设面向世界的妈祖文化小镇，打响妈祖文化国际品牌。打造全国文化强市，实施文化繁荣专项行动，全面完善公共文化设施，增加公共文化供给，弘扬传承莆仙戏等优秀传统文化，加大文物保护利用，大力发展文化产业，推动莆田文艺“走出去”，建成国家级公共文化服务体系示范区、全国地方戏曲保护传承示范区、国家级历史文化名城、中国文创之都、侨台联谊文化交流基地。打造国际旅游胜地，实施全域旅游专项行动，整合滨海文化生态旅游资源，打造多层次旅游产品体系，创新旅游业态，优化服务供给，打造“清新福建、妈祖圣地、美丽莆田”旅游品牌。

　　今后五年，我们要普惠民生，让人民生活更加幸福和谐。构筑人才“高地”，坚持刚性引才和柔性引才相结合，健全人才引育政策，强化人才服务保障，突出高层次人才和创新创业人才（团队）招引，打造多层次人才队伍，到2021年，各类专业技术人才超过16.5万人，高技能人才超过5万人。打造民生“福地”，实施民生改善专项行动，全面完成现行贫困人口脱贫、88个贫困村摘帽；建设教育强市，全面解决学前教育“入园难”，全面提升义务教育教学质量，全面普及高中阶段教育，创建3所以上高中省级示范校，大力发展高等教育和职业教育；打造健康中国的先行区，完善分级诊疗体系，建设一批养老设施、老年活动中心，每个县（区、管委会）完成2个以上医养结合试点，到2021年，千人均病床位达5.5张；完善公共体育设施，广泛开展全民健身，筹划举办品牌赛事，发展体育产业；实施就业促进计划和创业引领计划，实现稳定就业和更高质量就业，实施全民参保计划，推动社会保险实现全覆盖,全面提高保障水平，健全食品安全体系，创建国家食品安全示范城市。建设法治“善地”，实施社会治理专项行动，推进全域平安创建，加强重点领域风险管控，完善网格化治理运行机制和基层善治体系建设，最大限度减少社会不稳定因素，建设平安莆田、法治莆田、诚信莆田。

　　三、2017年工作安排

　　坚持稳中求进工作总基调，牢固树立和贯彻落实新发展理念，适应把握引领经济发展新常态，着力稳增长、促改革、调结构、惠民生、防风险，促进经济社会平稳健康发展。国民经济和社会发展的主要目标是：地区生产总值增长10%左右；一般公共预算总收入增长8%，地方一般公共预算收入增长7.5%；全社会固定资产投资增长15%；社会消费品零售总额增长12%；外贸出口总额增长3%，实际利用外资增长5%；居民人均可支配收入增长9%；城镇登记失业率控制在3%以内；居民消费价格涨幅控制在3%以内；有效落实节能减排降碳任务。

　　（一）推进农业供给侧结构性改革，做好“三农”工作

　　持续推进精准扶贫。实施扶贫脱贫攻坚工程，落实产业扶贫、就业扶贫、交通扶贫、低保兜底、生态补偿、医疗保障等帮扶措施。探索建立贫困对象增加财产性收益机制，新增公益性岗位1000个，完成脱贫8000人，造福搬迁3000人，改造农村危房400户，加大对仙游扶贫脱贫工作的支持力度。

　　积极发展特色现代农业。管好“米袋子”，落实惠农扶粮政策，稳定粮食播种面积和产量。丰富“菜篮子”，新增设施果蔬大棚3千亩，实施畜禽标准化生态养殖，新建楼房猪舍1万平方米、智能禽舍3万平方米。推广“好品牌”，推进“一区两园”建设，发展设施农业、观光农业、休闲农业，扩大“四大名果”、龙华薏米等地理标志农产品的品牌效应。开展第三次全国农业普查。

　　大力推进强村富民。优化提升村镇体系规划，全力推进美丽乡村建设，完善基础设施和公共服务配套，加快建设仙游-涵江环山区公路，续建新建农村公路100公里、陆岛交通码头13个，推进农村土地所有权、承包权、经营权三权分置，完成土地承包确权颁证，培育新型农业经营主体和服务主体，推动规模经营和农场化、公司化生产经营，发展“一村一品”、“一村一业”，壮大村级集体经济，引导农民创业增收。开展农村宅基地确权办证及抵押融资试点。

　　（二）推动产业转型升级，培育跨越发展新动能

　　持续发挥项目带动作用。突出观念更新，高端策划推进一批具有比较优势、符合莆田发展的大项目、好项目。突出精准招商、产业链招商和“零地招商”，着力引进一批重要莆商、知名跨国公司和行业龙头、企业总部或区域事业部、发达城市外迁优质企业，引进一批互补和联动类项目。落实“五个一批”项目推进工作机制，强化项目服务跟踪保障，确保480个重点项目完成年度投资980亿元，开工80个、竣工投产80个。

　　大力发展十大优势产业。实施绿色制造提升工程，加快壮大支柱产业，集聚发展新兴产业，积极发展建筑、能源产业。支柱产业，鼓励应用新工艺、新设备、新技术、新材料，加快创新转型。鞋服产业，重点建设共性技术研发平台、产业链服务平台，培育联盟品牌，推广石墨烯新材料应用，推动华峰纺织产业园、屹立智能化生产设备等投达产。化工（纺织化纤）新材料产业，重点加快CPL、PA6二期、赛得利三期等建设，延伸尼龙拉丝产业链，发展粘胶纤维和纺织新材料产业链。工艺美术产业，重点加快行业标准建设，建立检测鉴定中心，打造诚信品牌，提升区域品牌，继续办好艺博会和红木博览会，加快建设仙作工艺小镇、北高上塘珠宝银饰小镇。食品加工产业，重点加快英博雪津迁建、中粮两片罐、和润粮油等建设，成立“放心食品”产业联盟，发展精深加工产业链，创新品类、加强检测，提升产业竞争力。新兴产业，聚集前沿先导技术，坚持抓龙头、铸链条、建集群，推动倍增发展。医疗健康产业，成立医疗健康产业引导基金，组建投资开发集团，建设医疗健康产业园启动区，引导莆籍医疗企业回莆设立总部和医疗金融、信息服务机构，开工建设医疗器械耗材生产项目，筹建健康医疗大数据平台。电子信息产业，重点推动华佳彩一期、砷化镓等项目建成投产，建设第三代半导体整线设备项目，延伸发展玻璃基板、偏光片等新型显示上游配套及终端应用，培育发展集成电路装备、设计、封装和测试等产业链。高端装备产业，重点推动云度新能源汽车、海上风机制造等项目竣工投产，鞍钢冷轧复产，开工建设激光切割装备等项目，延伸发展电机、电控、汽车零部件、高端智能化数控机床等。海洋产业，重点发展海产品精深加工和远洋捕捞，推进南日鲍生态养殖、现代海洋渔业综合开发、海上风电场立体养殖示范区建设。建筑产业，重点落实建筑业发展扶持政策，加快培育引进特级、一级建筑企业及建筑业总部，开展装配式建筑施工及建筑垃圾循环利用试点。能源产业，重点加快推进LNG接收站二期、海上风电、哈纳斯LNG、抽水蓄能二期等项目建设，建成投产国投湄洲湾火电厂二期项目。

　　加快现代服务业提质增效。推动模式创新、跨界融合、多维拓展，发展生产性和生活性服务业。现代商贸业，发展特色街区、楼宇经济、社区商业和连锁经营，培育财务、咨询、工业设计等中介服务，鼓励工业企业工贸分离，加快海吉星农贸城和美食一条街等建设。新增规上服务业企业50家、限上商贸企业100家。软件和信息服务业，实施“互联网+”行动计划，筹建软件信息产业园，加快推进二手车第三方认证平台、联发软件产业园一期等项目。电子商务，重点打造跨境电商通关服务平台，建设跨境电商主题园区，扶持壮大普天药械网等细分领域平台，发展一批线上品牌，增加农村淘宝村级服务站100个，培育交易额超亿元企业6家，限上电商企业250家。金融服务业，切实防范和化解金融风险，建立诚信惩戒机制，维护金融诚信环境，大力引进培育金融机构，促进泉州银行开业、华林证券等7家证券公司入驻，扩大直接融资规模，推动10家以上后备企业在新三板、海交中心挂牌。房地产业，坚持“房子是用来住的，不是用来炒的”的定位，全面推行货币化安置，打通库存转换通道，推进商业办公用房去库存，科学安排土地供应，促进房地产市场平稳健康发展。

　　加速推动港口经济发展。加快港口基础设施建设，开工东吴作业区1-3#泊位、石门澳工业园区配套码头、江涵大桥等项目，推进莆头作业区3-6#泊位、联十一线、纵一线莆田段等建设，建成运营罗屿作业区40万吨大型散货码头、国投煤炭装船泊位、国投铁路支线、罗屿铁路支线等，开展向莆铁路货运专线、福莆宁城际轨道等前期工作。大力发展现代港口业态，积极培育新航线，争取瑞钢联铁矿石网上交易平台布点莆田，推进普洛斯二期、中粮、闽润等临港物流项目，建设煤炭、矿石、木材、油品等仓储中转基地，争取设立保税区、保税仓库，带动发展港航信息、贸易、金融等衍生增值服务。推进电子口岸“单一窗口”建设。

　　（三）统筹城乡发展，加快新型城镇化进程

　　完善城乡规划体系。健全完善城规委决策机制，完成“多规合一”工作，严格实行“三个一”规划审批，开展城市总规中期评估和“城市诊断”，编制和完成景观风貌、地下空间、名城保护及城区综合交通路网等专项规划，完成城市绿心总体规划和启动区详细规划，优化提升六大工业园区控制性规划，开展广化寺片区、木兰溪两岸等城市重点地段城市设计。

　　加强城市建设管理。推进一批片区改造更新，建设完整社区和高端社区，全面实施安置房建设专项清理行动，完成棚户区改造年度任务。着力解决“出行难”问题，建成荔塘快速路，续建提升木兰大道等六条东西干线，贯通改造涵港大道等五条南北干线，扩容建设黄石高速口等四个立交节点，坚持公交优先，新建新增一批公交场站、公交线路和停车泊位，全面建设公共自行车系统。完善功能配套，全面实施“十个一工程”项目，建设第五水厂、东圳水利枢纽引水配套工程，建成妈祖城水厂、市第三水厂、仙游第二水厂，综合治理下磨溪等城市内河和29处城区低洼地，建设“一轴两溪”重点地段夜景工程。深化“大城管”体制改革，推进“智慧城管”建设，严格城市综合管理考评，强化“两违”、道路交通、市容市貌等专项治理，完善常态化长效管理机制。

　　增强城镇集聚辐射。推进实施4个省级特色小镇“1+X”规划体系，基本形成榜头镇级小城市框架，深化全国建制镇示范试点、省市级小城镇综合试点，创建市级特色小镇，辐射带动一批幸福家园和新型农村社区建设，创出就近城镇化和就地市民化的莆田模式。

　　（四）保护改善环境，发展全域生态旅游

　　铁腕整治环境突出问题。加强重点行业企业大气污染物综合治理，全面淘汰黄标车及老旧车辆，完成大气污染防治年度目标。加强水源地、流域和农村水环境综合治理，完成城区4个片区污水管网改造、16条黑臭水体整治和污水源整改任务，完成木兰溪沿线乡镇、省级以上试点镇和六大工业园区的污水收集，饮用水源水质达标率、流域和内河水质功能达标率控制在省下达指标内。强化城乡生活垃圾收集处理，建成市垃圾焚烧发电厂三期扩建项目，开工建设仙游垃圾焚烧发电厂、厨余、危废垃圾处理等项目。全面开展石材加工、废旧塑料加工、粘土机砖瓦窑、塑料拉丝编织和翻砂铸造生产五项专项治理，推行企业生产清洁化、循环化，有效整治工业污染源，进一步整治畜禽养殖、农药化肥使用等农业污染源，完成土壤污染防治年度任务。

　　加强生态环境保护和修复。建设木兰溪防洪工程、乌溪水库、西音水库等一批水利项目，推动东圳水库饮用水源、城市绿心保护立法，实施流域生态保护补偿全覆盖，开展绿色发展绩效评价考核和自然资源资产离任审计，开展重点生态区位商品林赎买试点，完成重点生态区位林分修复7000亩，建设木兰溪源森林公园、木兰陂水利公园、秀屿土海湿地公园，续建新建城市公园、“口袋”公园和绿道。开展近岸海域环境保护、污染防治，实施海岸带生态环境修复工程。

　　大力发展生态旅游。制定全域旅游行动计划实施方案，加快旅游交通导视和配套服务设施体系建设，提升景区服务功能，加强旅游线路运作、品牌推介和市场监管，扶持发展重点旅游企业。加快建设妈祖文化小镇，开工建设湄洲岛游客码头，推动湄洲岛、九鲤湖创建国家5A级景区，菜溪岩、大蚶山创建国家4A级景区。提升南少林等景区品质，开发西苑仙水洋、庄边宁里旅游资源，创建休闲集镇2个、特色村5个、星级乡村旅游接待单位5家，新增旅游观光工厂2个、旅游景点线路6条。

　　（五）坚持改革开放创新，激活跨越发展新活力

　　深化重点领域改革。推进“放管服”改革，完成市县乡政府权责清单融合统一，推行“互联网+政务服务”，完善项目并联审批办法，拓展“一窗受理”范围，实现“双随机一公开”监管全覆盖，完成行政服务标准化试点验收。推进商事制度改革，实现企业电子化登记，推进个体工商户营业执照和税务登记证“两证整合”。深化投融资体制改革，推进预决算公开，公布政务服务、政府购买服务目录，推广PPP模式。启动市属国有企业二次重组整合，实行分类管理，推动股权多元化。完善产权保护制度，加强各种所有制经济产权保护。

　　加大双向开放合作。实施“走出去”战略，鼓励企业“抱团出海”，开拓南亚、西亚、非洲东海岸等新兴市场，落实外贸出口扶持政策，稳定出口增长，推动先进技术设备、关键零部件进口。深化莆台交流合作，开展对台客运码头前期工作，发展对台小额贸易，拓展莆港澳金融、物流、专业服务、文化、旅游等领域合作，加强与侨界紧密合作。建成通关联检大楼，争取秀屿港口岸扩大对外开放获批。复制推广福建自贸区成熟改革创新举措，承接政策外溢和辐射带动效应，深化福莆宁岚同城化合作，推动设施互通、产业互补、资源共享，密切闽东北、闽浙赣皖等地区协作，扎实做好对口帮扶工作。争取与印尼棉兰、澳大利亚朗塞斯顿缔结友好城市。

　　健全创新服务体系。设立产业研发专项基金，推动政产学研深度合作，加快建设中科院科技服务网络计划中心莆田分中心，建成中科院海西研究院莆田中心，新增5个省级以上工程技术中心、重点实验室。设立创投引导基金、专利发展基金，引导全社会加大科技创新、成果转化投入，设立3600万元人才发展基金，开展“智汇莆田”专项行动，引进20个以上高层次团队，聘任一批拔尖人才、高端人才担任政府顾问，吸引国内外包括莆籍人才担任客座教授、交流学者、技术顾问，设立专家工作站、博士后工作站，建立实训基地，培养应用、研发人才。搭建企业家培训对话平台、信息共享平台，加强培训，提升素质。

　　（六）弘扬莆仙优秀文化，推动文化强市建设

　　推进妈祖文化品牌建设。实施妈祖文化弘扬工程，举办第二届世界妈祖文化论坛，建设论坛永久会址，举办妈祖春秋祭典、海峡论坛·妈祖文化周、中国·湄洲妈祖文化旅游节等活动。联合推动“海丝”申遗，设立妈祖文化基金，加强妈祖文化研究，扩大妈祖文化交流和传播。

　　增加公共文化服务供给。全面投用市“三馆一宫”、规划展示馆和报业采编中心，加快推进市档案馆迁建工程，筹建市美术馆，推进县区“三馆”和乡镇（街道）、村居（社区）综合文化服务设施建设，免费开放公共文化设施。实施一批文物修复工程，开发一批历史文化景点，推进“六街八景”和传统古村落保护利用，挖掘莆仙人文资源，传承家风家训，讲好莆田故事，广泛开展群众性文化活动。

　　大力发展文化产业。扩大和引导文化消费，设立文艺精品项目库，出版一批文学、社科作品，扶持莆仙戏剧本创作和演出业，引导现代工艺设计融入传统文化元素，提升传统工艺产品附加值，发展数字动漫、电子出版、创意设计、影视演艺等前沿文化产业，扶持新兴文化企业。

　　（七）突出公平公正普惠，扩大人民群众获得感

　　发展优质均衡教育。实施教育强市提升工程。扩大普惠性学前教育，落实小区配套幼儿园建设，新建公办幼儿园，学前三年入园率达98%。实施“全面改薄”，促进扩容提质，新建扩建城区小学、初中学校15所，加快莆田一中、莆田八中新校区前期工作，义务教育巩固率达98%，高中阶段毛入学率达95.6%。实施十二年免费特殊教育，积极发展继续教育，支持和规范民办教育发展，推动创办国际学校，大力发展现代职业教育，加快莆田学院、湄洲湾职业技术学院迁建项目建设。

　　推动健康莆田建设。实施健康莆田提升工程，加大优质医疗资源供给，建成市第一医院门诊大楼、市口腔医院、市中医院一期，迁建市皮肤病防治医院，启动市第一医院和莆田学院附属医院独立专科医院建设，加大妇产科、儿科投入，2个乡镇卫生院达到二级医院规模，新增病床位1000张，千人均病床位达5张。深化医药体制改革，完善医保便民支付方式，强化大病补充医疗保障，开展分级诊疗，支持民营医院规范发展。做好计划生育工作，工作重点由管理向服务转变。加强城乡公共体育设施建设，广泛开展全民健身活动，办好市第四届运动会。

　　提高社会保障水平。实施社会保障提升工程。落实积极就业政策，加强基层就业社会保障服务，新增城镇就业1.7万人，做好军转干部和退役士兵接收安置。实施全民参保登记计划，加大医保投入，城乡基本医疗参保人数超过300万人。加快建立以居家养老服务为主，养老机构为补充，医养结合的多层次可持续的社会养老服务体系，新建20个养老设施和一批老年文化教育活动场所，城乡居民基本养老保险参保人数超过154万人。鼓励社会力量参与发展残疾人、社会福利和慈善事业，加大社会救助力度，关爱留守儿童、空巢老人和务工人员随迁子女。办好19项市委市政府为民办实事项目。

　　加强和创新社会治理。实施全域平安创建工程，开展安全生产、食品安全、危险物品等整治，做好应急处置，推动国家防震减灾示范城市创建工作。实施社会和谐建设工程，深化阳光信访、责任信访、法治信访，加强矛盾纠纷多元化解、法律援助和司法救助。实施基层治理提升工程，加强打防管控，完善网格化治理，城区视频监控覆盖率达100%、农村超过70%，平安和谐村居达标率超过40%。开展“七五”普法，增强全民法治意识，加强社会诚信建设，落实民族宗教政策，发挥好工会、共青团、妇联、工商联等人民团体作用，争创全国文明城市。推进军民融合深度发展，争创全国双拥模范城“六连冠”。

　　四、从严从实加强政府自身建设

　　坚决维护以习近平同志为核心的党中央权威，牢固树立“四个意识”，努力打造人民满意的法治政府、创新政府、廉洁政府和服务型政府。

　　我们要高点站位，更新观念，增强本领，不断提升政府创新力。深化对经济新常态、发展新理念的认识和理解，围绕“再上新台阶、建设新福建”和我市今后五年的发展大局，解放思想，破除因循守旧的思维定势，树立“弯道超车”“励志超越”的理念，以国际视野和战略思维谋发展干事业。坚持问题导向，围绕产业转型、城市建设、民生改善、风险防控等工作重点，借鉴先进地区好机制好办法，跟踪研究新知识、新技术、新模式，增强新本领，探索新途径，创造新业绩，依靠创新赢得主动、赢得优势、赢得未来。

　　我们要勇于担当，马上就办，真抓实干，不断提升政府执行力。新常态下抓发展，最需要的就是担当精神。我们要提振全市上下干事创业的精气神，力戒“不冷不热、不急不忙、不痛不痒”的工作态度，以踏石留印、抓铁有痕的勇气，落实“马上就办”，对谋定的事，说了就干、干就干好，干出水平、干出成效，树立敢担当、能担当、会担当的新形象。建立干部正向激励和容错纠错机制，旗帜鲜明地为担当者担当、为负责者负责，克服庸政、懒政、怠政，形成人人想干事、敢干事、会干事的环境和氛围。

　　我们要依法行政，科学决策，信守承诺，不断提升政府公信力。坚持把法治精神、法治思维、法治方式融入政府工作全过程。自觉接受人大及其常委会的法律监督和工作监督、政协的民主监督、社会公众监督和舆论监督，加强与法院、检察院良性互动衔接，落实政府法律顾问制度。强化政务公开和政府信息公开，健全公众参与、专家论证、风险评估、集体研究的重大决策机制。信守政府承诺，力戒“新官不理旧账”，以政府诚信带动社会诚信。

　　我们要以上率下，政府清廉，干部清正，不断提升政府向心力。全面落实从严治党主体责任，严明政治纪律和政治规矩，加强党风廉政建设和反腐败工作，按本色做人、按角色做事，强化行政监察和审计监督，以铁的纪律维护良好政治生态。着力构建“亲”“清”新型政商关系，打造一流营商环境，真诚坦荡同民营企业家接触交往，真心实意为企业排忧解难，精心呵护好企业家和人才队伍，让创新活力在莆田竞相迸发，让创业激情在莆田澎湃涌动！

　　各位代表，让我们紧密团结在以习近平同志为核心的党中央周围，在省委省政府和市委的坚强领导下，以更加饱满的热情、更加昂扬的斗志、更加务实的作风，不忘初心，砥砺奋进，跨越赶超，为“坚持绿色发展，打造宜居港城、建设美丽莆田，创建美丽中国的示范区”而努力奋斗，以优异的成绩迎接党的十九大胜利召开！
